# Supplementary material for: Enterococcus faecalis modulates phase variation in Clostridioides difficile
Source: J Bacteriol. 2025 Dec 23;208(1):e00374-25. doi: 10.1128/jb.00374-25 (PMC12826044; doi:10.1128/jb.00374-25)
Supplement: Supplemental figures and tables — Figures S1 to S4, and Tables S1 and S2. [file jb.00374-25-s0001.pdf]

**SUPPLEMENTAL MATERIAL**

# *Enterococcus faecalis* modulates phase variation in *Clostridioides difficile*

Ashley S. Weiss[1,2¶], Jilarie A. Santos-Santiago[3¶], Orlaith Keenan[1,2], Alexander B. Smith[1,2], Montana Knight[4], Joseph P. Zackular[1,2,5,&*], Rita Tamayo[3,&*]

[1] Division of Protective Immunity, Children's Hospital of Philadelphia, Philadelphia, Pennsylvania, USA

[2] Department of Pathology and Laboratory Medicine, Perelman School of Medicine, University of Pennsylvania, Philadelphia, Pennsylvania, USA

[3] Department of Microbiology and Immunology, University of North Carolina Chapel Hill, Chapel Hill, North Carolina, USA

[4] Department of Biomedical and Health Informatics, Children's Hospital of Philadelphia, Philadelphia, Pennsylvania, USA

[5] Center for Microbial Medicine, Children's Hospital of Philadelphia, Philadelphia, Pennsylvania, USA

* Corresponding authors

E-mails: rita_tamayo@med.unc.edu (RT), joseph.zackular@pennmedicine.upenn.edu (JPZ)

[¶] These authors contributed equally to this work.

[&] These authors also contributed equally to this work.

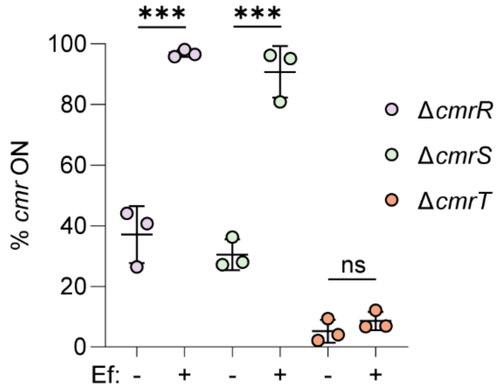


**Supplemental Figure 1. *C. difficile* requires *cmrT* for the *E. faecalis*-mediated increase in *cmr*-ON.** Quantitative PCR of *cmr* switch orientation in macrocolonies after 5 days of growth of Δ*cmrR* Cd, Δ*cmrS* Cd, and Δ*cmrT* Cd in monocultures or dual cultures with Ef at a 1:1 ratio. Data from 3 biological replicates were analyzed and presented as means and standard error. ns = not significant, *** $p < 0.001$, unpaired t-tests.

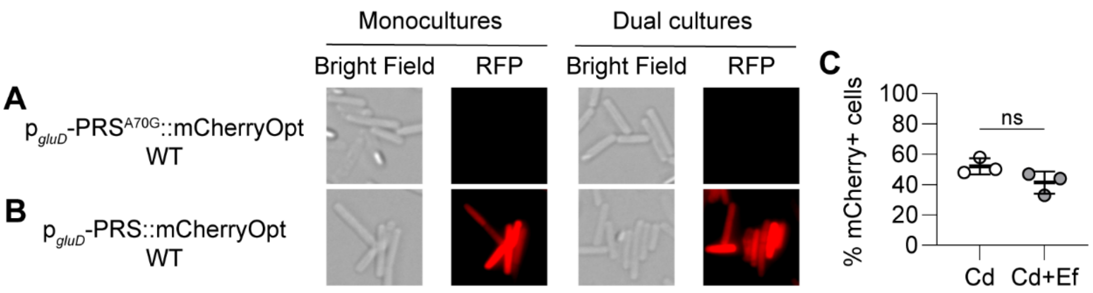


**Supplemental Figure 2. *E. faecalis* does not alter global c-di-GMP levels in culture with *C. difficile*.** Representative micrographs of reporter strains. *C. difficile* reporter strains (**A**) $p_{gluD}$-PRS$^{A70G}$::mCherryOpt (negative control), and (**B**) $p_{gluD}$-PRS::mCherryOpt, were grown for 7 days in monocultures or dual cultures with *E. faecalis* at a 1:1 ratio before preparing micrographs and imaging at 100X. (**C**) Percentage of mCherryOpt-expressing cells. Data from 3 biological replicates were analyzed and presented as means and standard error. ns = not significant, unpaired t-test.

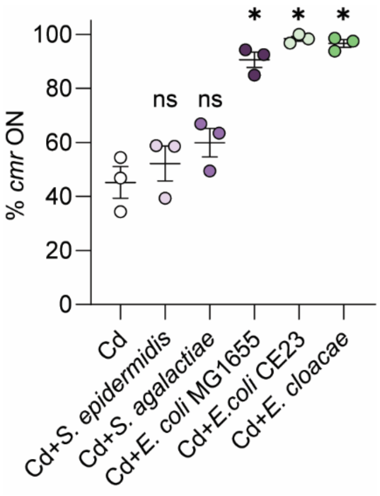


**Supplemental Figure 3. Certain enteric bacterium increase *cmr*-ON in *C. difficile.*** Quantitative PCR of *cmr* switch orientation in macrocolonies after 5 days of growth with comparisons made to Cd. Macrocolonies were Cd monocultures or dual cultures with Cd and *S. epidermidis*, *S. agalactiae*, *E. coli* MG1655, *E. coli* CE23, or *E. cloacae* at a 1:1 ratio. Data are presented as means and standard error, with symbols representing biological replicates. ns = not significant, * $p < 0.05$, Brown-Forsythe and Welch ANOVA tests.

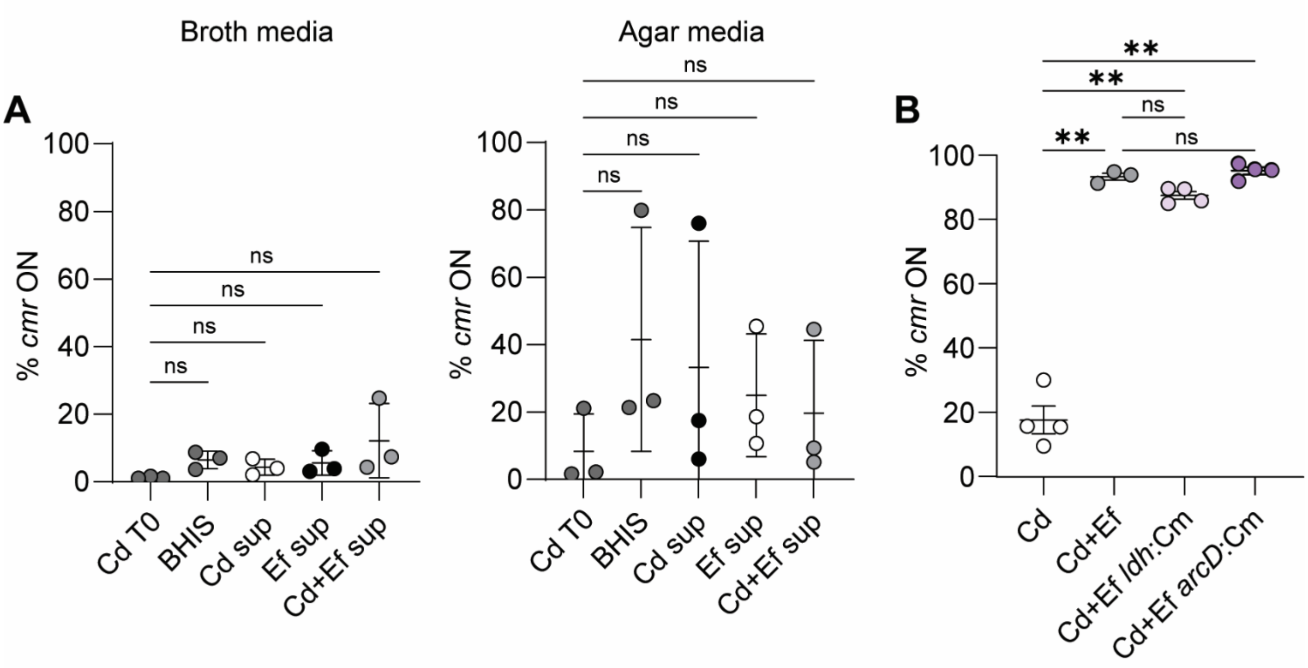


**Supplemental Figure 4. *E. faecalis* broth supernatants and alterations in pH do not enrich for *cmr*-ON in *C. difficile.*** (**A**) Quantitative PCR of *cmr* switch orientation in Cd R20921 grown in broth media for 5 days or on agar media overnight. The orientation of the *cmr* switch was measured in Cd before it was added to other culture conditions (Cd T0). Broth and agar media were supplemented with BHIS broth or supernatants (sup) from Ef or Cd monocultures, or Cd+Ef dual culture. Cd alone was grown in each of these conditions, and *cmr* switch orientation was measured relative to Cd T0. (**B**) Quantitative PCR of *cmr* switch orientation in Cd R20921 macrocolonies or dual culture macrocolonies with Ef, Ef *ldh*:Cm, or Ef *arcD*:Cm after 5 days of growth. Data are presented as means and standard error, with symbols representing biological replicates. ns = not significant, ** $p < 0.01$, Brown-Forsythe and Welch ANOVA tests.

**Table S1. Strains and plasmids used in this study.**

| Lab Notation* | Strain name | Description | Reference |
|---|---|---|---|
| ***C. difficile* strains** | | | |
| RT273, Z033 | *C. difficile* R20291 | Ribotype 027 strain, smooth colony isolate (BioSample GCA_000027105) | (13) |
| RT3332 | *C. difficile* Δ*fliC* | R20291 with in-frame deletion of *fliC* | This work |
| RT1341 | *C. difficile* Δ*pilA* | R20291 *pilA1*::*ermB*; Targetron Insertion | (19) |
| RT1566 | *C. difficile* Δ*sigD* | R20291 *sigD*::*ermB*; Targetron Insertion | (20) |
| RT947 | *C. difficile* Δ*pilB* | R20291 *pilB1*::*ermB*; Targetron Insertion | (17) |
| RT2256, Z036 | *C. difficile* Δ*cmrR* | R20291 with in-frame deletion of *cmrR* | (7) |
| RT2807, Z209 | *C. difficile* Δ*cmrS* | R20291 with in-frame deletion *cmrS* | (7) |
| RT2257, Z037 | *C. difficile* Δ*cmrT* | R20291 with in-frame deletion of *cmrT* | (7) |
| RT2296, Z038 | *C. difficile* Δ*cmrR*Δ*cmrT* | R20291 with in-frame deletions of *cmrR* and *cmrT* | (7) |
| RT2406, Z210 | *C. difficile cmr*Δ3-ON | R20291 with *cmr* switch phase-locked in ON orientation due to deletion of three nucleotides in the right inverted repeat | (9) |
| RT2395, Z211 | *C. difficile cmr*Δ3-OFF | R20291 with *cmr* switch phase-locked in OFF orientation due to deletion of three nucleotides in the right inverted repeat | (9) |
| RT2816 | *C. difficile* $P_{gluD}$-PRS::mCherryOpt | R20291 with c-di-GMP reporter consisting of *gluD* promoter, *pilA1* c-di-GMP riboswitch, and codon-optimized mCherry | (23) |
| RT2813 | *C. difficile* $P_{gluD}$-PRS$^{A70G}$::mCherryOpt | R20291 with c-di-GMP reporter containing a mutation (A70G) in the riboswitch rendering it blind to c-di-GMP | (23) |
| RT1065 | *C. difficile* UK1 | Ribotype 027 | (24,25) |
| RT3321 | *C. difficile* M120 | Ribotype 078 | (26) |
| RT1357 | *C. difficile* ATCC BAA-1875 | Ribotype 078 | ATCC |
| RT1124 | *C. difficile* 630 | Ribotype 012 strain | (7) |
| RT1125 | *C. difficile* VPI 10463 | Ribotype 087 strain | (27) |
| RT1358 | *C. difficile* ATCC 43598 | Ribotype 017 strain | ATCC |
| ***Enterococcus* strains** | | | |
| RT2378, Z002 | *E. faecalis* OG1RF ATCC 47077 | "EF OG1RF" | ATCC |
| Z007 | *E. faecalis* V583 VRE ATCC 700802 | "Ef V583 VRE" | ATCC |
| AW69 | *E. faecalis* IGram | "Ef IGram"; isolated from clinical sample in the Igram study, IGram 142 from 1mo. infant | (44,45) |

| | | | |
|---|---|---|---|
| ABS055 | *E. faecalis* Pedscom PC15 | "Ef Pedscom"; strain from Pedscom defined microbial consortia shared by CHOP | (29) |
| Z167 | *E. faecalis ldh:*Cm | "Ef *ldh:*Cm"; *ldh* transposon mutant strain | (32) |
| ABS078 | *E. faecium* VRE ATCC 700221 | "Efm VRE" | ATCC |
| Z221 | *E. faecium* DVT705 VRE | "Efm DVT705"; clinical isolate from the University of Pittsburgh | (30) |
| AW65 | *E. faecium* IGram | "Efm IGram"; isolated from clinical sample in the IGram study, IGram 033 from 12mo. infant | (44,45) |
| Z020 | *E. faecalis arcD:*Cm | "Ef *ldh:*Cm"; *arcD* transposon mutant strain | (11,32) |
| AW25 | *E. durans* | "Ed"; isolated from clinical sample in DYNAMIC study, DYNAMIC 1-16 | (43) |
| **Enteric bacterial strains** | | | |
| Z094, AW50 | *E. coli* MG1655 | Substrain of E. coli K-12 from UPenn | NCBI: txid511145 |
| Z178, AW54 | *E. coli* CE23 | Enteropathogenic *E. coli* clinical strain from University of Pennsylvania | |
| AW43 | *S. agalactiae* CJB111 | "*S. agalactiae*"; strain from the University of Colorado Anschutz | NCBI: txid342617 |
| Z227, AW38 | *S. epidermidis* ATCC 14490 | | ATCC |
| Z074 | *E. cloacae* ATCC 13047 | | ATCC |

* RT notation indicates strain in R. Tamayo lab collection; Z/AW notation indicates strain in J. Zackular lab collection.

**Table S2. Primers used in this study.**

| Lab Notation | Primer Name | Sequence |
|---|---|---|
| R850 | *rpoC*-F | CTAGCTGCTCCTATGTCTCACATC |
| R851 | *rpoC*-R | CCAGTCTCTCCTGGATCAACTA |
| R2298 | *cmrR*-F | AAGAAAAAGTTCGGGGATTTTTAGC |
| R2299 | *cmrR*-R | CGCTGAAAACTTTAACACATTAGGA |
| R2537 | *cmrT*-F | GACAAGGATAATTGCC |
| R2538 | *cmrT*-R | CCATCACCATCAGTTAG |
| R2539 | *cmrS*-F | GATAGATGACTGGGAG |
| R2540 | *cmrS*-R | CGATAAGTAGCATTCCC |
| R2270 / R2271 | cmr ON F/R | GGAGATATATGGAGTTAGTGGTGCAA / CTAGCCAATAGACAAGTTTCTAGAAAAATA |
| R2271 / R2272 | cmr OFF F/R | CTAGCCAATAGACAAGTTTCTAGAAAAATA / GAACAATTCTTGAATATTGTATTGAACATTAAGA |
| R3863 | fliC-F1 | gtccattgatttctttcagtttcggatccGAAGAAGCTGTTAAAAATATGCAAGC* |
| R3864 | fliC-R1 | CTTCTATATCCAATATACAAAATTATTTTAT CCGGACAAGATGGTACATAACTTTATAC |
| R3865 | fliC-F2 | GTATAAAGTTATGTACCATCTTGTCCGGAT AAAATAATTTTGTATATTGGATATAGAAG |
| R3866 | fliC-R2 | gtgacgtcgactctagaggatccCGAATCTATACTATTTTCATACTTCC* |
| R3867 | fliC-F0 | GCAAAGTTGACTTCTGTCACTG |
| R3868 | fliC-R0 | CTGAAAATAAACTATTTGCTTGTGTAC |

* Lowercase text indicates complementarity to vector.
